# Sexuality in ADHD: empirical data concerning hypersexual and paraphilic fantasies and behaviors in adults with ADHD

**DOI:** 10.1192/j.eurpsy.2022.725

**Published:** 2022-09-01

**Authors:** P. Gregório Hertz, D. Turner, W. Retz

**Affiliations:** University Medical Center Mainz, Department Of Psychiatry And Psychotherapy, Mainz, Germany

**Keywords:** ADHD, hypersexuality, paraphilia

## Abstract

**Introduction:**

ADHD is a neurodevelopmental disorder displaying inattention, hyperactivity, and impulsivity as core symptoms. It can affect several areas of life including sexual health. Clinicians have often made assumptions concerning the bound of specific ADHD symptoms affecting sexual desire by increasing its frequency and intensity. Yet, there is still a lack of knowledge about the comorbidity between ADHD, hypersexuality, and paraphilias. A recent literature review could show that some individuals who suffer from ADHD report about hypersexual and paraphilic fantasies and behaviors, but as far as we know, no clear empirical data has emerged supporting the idea that hypersexuality and paraphilias are more frequent in individuals with ADHD.

**Objectives:**

The present investigation aimed to compare several sexuality related aspects between individuals with and without ADHD.

**Methods:**

Therefore, we designed an extensive online survey based on established questionnaires, such as the Hypersexual Behavior Inventory (HBI). The survey was implemented in a outpatient sample, ADHD specific fora as well as other general online channels.

**Results:**

In total, N = 238 individuals participated in the survey (n = 160 with ADHD). Thereby, individuals with ADHD reported significantly more often about a wide range of hypersexual fantasies and behaviors in comparison to individuals without ADHD. Furthermore, individuals with ADHD reported significantly more often about paraphilic fantasies and behaviors including fetishistic and sadistic sexual fantasies. No differences were found concerning other paraphilias. Further results regarding other facets of sexuality, such as sexual orientation, are to be presented and discussed.

**Conclusions:**

The present study contributes to closing the knowledge gap regarding sexuality in individuals with an ADHD.

**Disclosure:**

No significant relationships.

